# Nanozyme for tumor therapy: Surface modification matters

**DOI:** 10.1002/EXP.20210005

**Published:** 2021-09-01

**Authors:** Guoheng Tang, Jiuyang He, Juewen Liu, Xiyun Yan, Kelong Fan

**Affiliations:** ^1^ CAS Engineering Laboratory for Nanozyme, Key Laboratory of Protein and Peptide Pharmaceutical, Institute of Biophysics Chinese Academy of Sciences Beijing 100101 P. R. China; ^2^ University of Chinese Academy of Sciences Beijing 101408 P. R. China; ^3^ Department of Chemistry, Waterloo Institute for Nanotechnology University of Waterloo Waterloo Ontario N2L 3G1 Canada; ^4^ Nanozyme Medical Center, School of Basic Medical Sciences Zhengzhou University Zhengzhou 450001 P. R. China

**Keywords:** cytotoxicity, Fe_3_O_4_ nanozymes, peroxidase‐like activity, surface modification, tumor catalytic therapy

## Abstract

As the next generation of artificial enzymes, nanozymes have shown unique properties compared to its natural counterparts, such as stability in harsh environment, low cost, and ease of production and modification, paving the way for its biomedical applications. Among them, tumor catalytic therapy mediated by the generation of reactive oxygen species (ROS) has made great progress mainly from the peroxidase‐like activity of nanozymes. Fe_3_O_4_ nanozymes, the earliest type of nanomaterial discovered to possess peroxidase‐like activity, has consequently received wide attention for tumor therapy due to its ROS generation ability and tumor cell killing ability. However, inconsistent results of cytotoxicity were observed between different reports, and some even showed the scavenging of ROS in some cases. By collectively studying these inconsistent outcomes, we raise the question whether surface modification of Fe_3_O_4_ nanozymes, either through affecting peroxidase activity or by affecting the biodistribution and intracellular fate, play an important role in its therapeutic effects. This review will go over the fundamental catalytic mechanisms of Fe_3_O_4_ nanozymes and recent advances in tumor catalytic therapy, and discuss the importance of surface modification. Employing Fe_3_O_4_ nanozymes as an example, we hope to provide an outlook on the improvement of nanozyme‐based antitumor activity.

## Graphical abstract

The effects of surface modification on the antitumor activity of nanozymes are summarized in three ways: (1) directly or indirectly affecting the catalytic activity of nanozymes; (2) regulating the biodistribution of nanozymes; (3) alternating the intracellular fate of nanozymes.

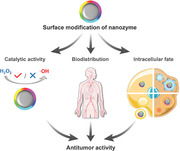

## 1 INTRODUCTION

Iron oxide nanoparticles (IONPs), which include Fe_3_O_4_ NPs, γ‐Fe_2_O_3_ NPs, and α‐Fe_2_O_3_ NPs, have shown promising application prospects in the diagnosis and therapy of diseases due to their unique properties at nanoscale.^[^ ^1^ ^]^ For example, their superparamagnetism is suitable for magnetic resonance imaging (MRI), and their photothermal conversion ability allows for photothermal therapy.^[^ ^2^ ^]^ Accompanied with high biosafety and biocompatibility, IONPs became the first generation of inorganic nanomaterials approved by the US Food and Drug Administration (FDA).^[^ ^3^ ^]^

**FIGURE 1:**
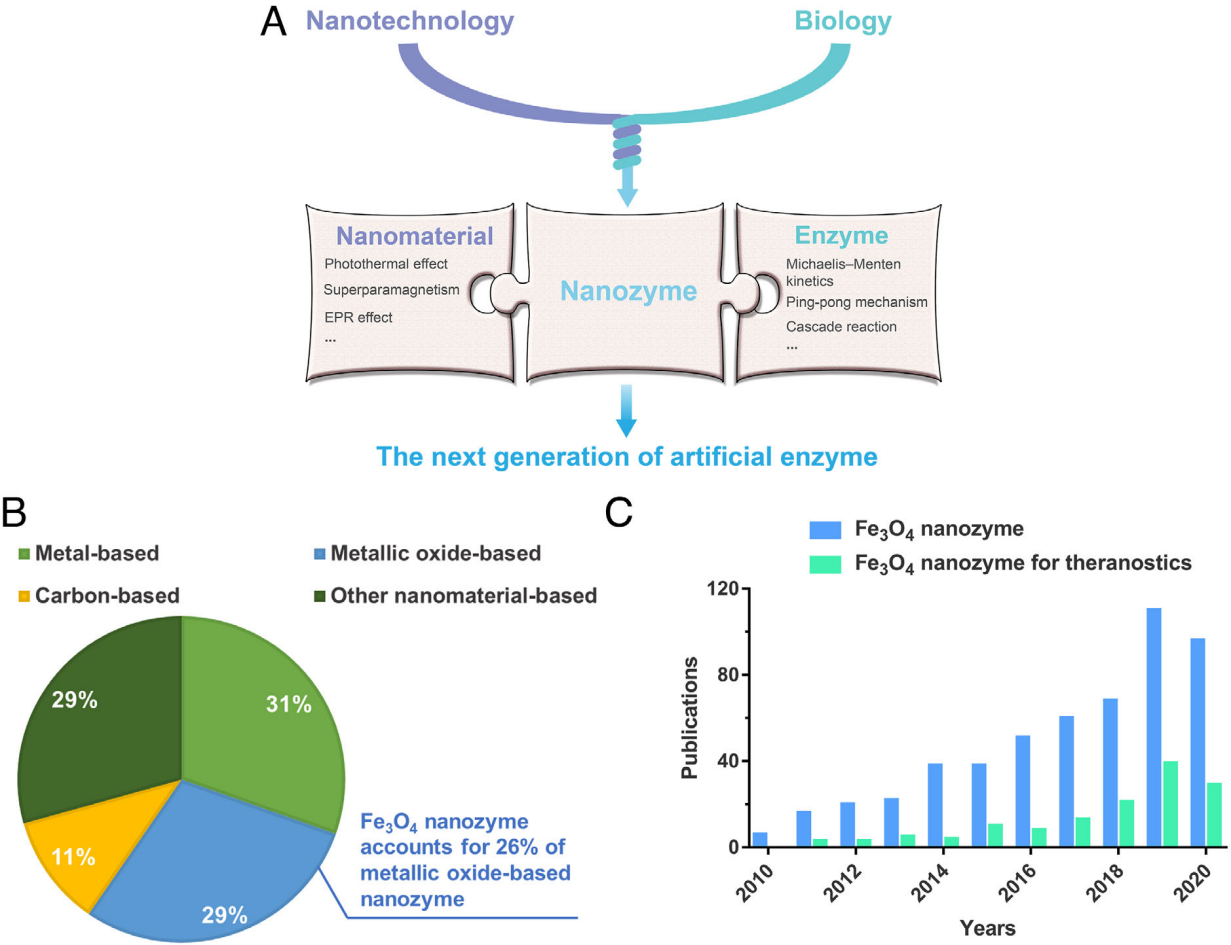
Introduction of nanozyme and Fe_3_O_4_ nanozyme. (A) Schematic illustration of relationship between nanozyme nanomaterial and enzyme. (B) Proportion of each material type of nanozyme and proportion of Fe_3_O_4_ nanozyme in metallic oxide nanozyme represented in pie chart (to January 1, 2021). Data based on Web of Science. (C) Fe_3_O_4_ nanozyme‐related publications by year (to January 1, 2021). Data based on Web of Science

Surprisingly, apart from the above physical properties, a novel biological property of IONPs—enzyme‐like activity was found in 2007 and subsequently the concept of nanomaterials with enzyme‐like properties was coined nanozyme.^[^ ^4^ ^]^ As an emerging field bridging nanotechnology and biology, nanozyme research aims to integrate properties of nanomaterials and gain inspirations from natural enzymes to become the next generation of artificial enzymes (Figure 1A). Up to now, the enzyme activities that nanozymes can mimic, from four types of oxidoreductase (peroxidase, oxidase, catalase, and superoxide dismutase) activities to other types of enzyme activities (e.g., hydrolase family and lyase family), showing a continuous growing trend. In terms of the materials, it mainly includes metal‐based, metallic oxide‐based, carbon‐based, and other materials‐based nanozymes.^[^ ^5^ ^]^ Although nanozymes and natural enzymes are significantly different in structure, they share many features in common. For example, the reactions of most nanozymes follow the Michaelis–Menten kinetics and the ping‐pong mechanism. Furthermore, the active centers or electron‐transport structures of nanozymes usually are similar to natural enzymes.^[^ ^6^ ^]^ Notably, compared with natural enzymes, nanozymes have several advantages in practical applications, including robustness in harsh conditions, low cost, ease of production and modification, and suitable for long‐term storage. Consequently, impressive progresses based on various nanozymes have been made in the recent years in extensive applications, from biosensing/bioanalysis and environmental monitoring/protection (in vitro) to disease theranostics (in vivo).^[^ ^4c^ ^]^ Wherein, Fe_3_O_4_ nanozymes, one of the first discovered and the most studied nanozymes (Figure 1B), demonstrate promising potential to integrate the advantages of nanozymes as well as the biosafety of IONPs in the context of theranostics (Figure 1C).

Utilizing its peroxidase‐like (POD‐like) activity, Fe_3_O_4_ nanozymes have been applied in various strategies for tumor catalytic therapy and have achieved positive outcomes^[^ ^7^ ^]^ owing to the elevation of reactive oxygen species (ROS) levels, which killed tumor cells through the increase of lipid peroxidation and the disfunction of proteins and chromosomes.^[^ ^8^ ^]^ In addition, increasing evidence supported that the POD‐like activity could be detected in FDA approved IONPs such as ferumoxytol and ferucarbotran,^[^ ^9^ ^]^ providing feasibility for as a tumor‐killing drug.

However, many studies showed opposite results; researchers found that Fe_3_O_4_ nanoparticles (Fe_3_O_4_ NPs), did not show any sign of POD‐like activity, causing negligible cytotoxicity to cancer cells.^[^ ^10^ ^]^ In some cases, Fe_3_O_4_ nanozymes even showed catalase‐like (CAT‐like) activity, which led to the clearance of intracellular ROS.^[^ ^8^, ^11^ ^]^

There are plenty of reasons for these inconsistent outcomes. For instance, the catalytic activity of Fe_3_O_4_ nanozymes can be modulated by size, morphology, and dopant, in correspond to the variation of ROS production.^[^ ^4^, ^7^, ^12^ ^]^ In addition, size, morphology, as well as the administration route (e.g., subcutaneous or intravenous injection) affect different biological effects (e.g., biodistribution and cellular uptake), thus increasing the accumulation in target sites or leading to undesired side effects.^[^ ^13^ ^]^

Herein, we believe that it is necessary to discuss the effects of surface modification, by taking Fe_3_O_4_ nanozyme as an example. Essentially, surface modification has brought encouraging progress to tumor therapy, which mainly lies in increasing the stability and biocompatibility of nanoparticles as well as providing sites for loading drugs.^[^ ^14^ ^]^ After collecting and comparing the studies with inconsistent outcomes, we think surface modification also plays an important role in affecting the antitumor activity of Fe_3_O_4_ nanozymes, which in general could be classified into four categories: (1) directly affecting the catalytic activity of Fe_3_O_4_ nanozymes; (2) indirectly affecting the catalytic activity of Fe_3_O_4_ nanozymes via the interaction between biological environment; (3) regulating the biodistribution of Fe_3_O_4_ nanozymes after intravenous injection; and (4) alternating the intracellular fate of Fe_3_O_4_ nanozymes (the already known fates include but not limited to lysosome/cytosol localization, autophagy, and exocytosis). Therefore, systematically summarizing and exploring the underlying effects of surface modification is beneficial to improve the therapeutic efficacy of Fe_3_O_4_ nanozyme‐based tumor catalytic therapy.

In this review, we first introduce the catalytic mechanisms of Fe_3_O_4_ nanozymes and update advances of Fe_3_O_4_ nanozyme‐based tumor catalytic therapy in brief. Then, the importance of surface modification was reviewed. Subsequently, the challenges of the uncertainties in existing research are discussed. With the hope of improving the antitumor activity of Fe_3_O_4_ nanozymes, we also point out some possible strategies through surface modification.

## 2 Fe_3_O_4_ NANOZYMES FOR TUMOR CATALYTIC THERAPY

### 2.1 Catalytic mechanisms of Fe_3_O_4_ nanozymes

Since the term nanozyme was first established, quest for its catalytic mechanisms has become one of the primary tasks in this emerging research area. Especially, catalytic mechanisms of Fe_3_O_4_ nanozymes is one of the most extensively studied. The enzyme‐like activities of Fe_3_O_4_ nanozymes comprise peroxidase‐like (POD‐like) activity and catalase‐like (CAT‐like) activity, normally determined by the environmental pH. Fe_3_O_4_ nanozymes exhibit POD‐like activity under acidic pH (3‐6.5) to catalyze reactions similar to natural peroxidase, in which H_2_O_2_ is decomposed to generate free radicals (mainly hydroxyl radicals) and then the hydrogen donor is oxidized to form the oxidized donor and water (Equation 1, where “AH” refers to hydrogen donor and A refers to oxidized donor). While under neutral and basic pH (7–10), the CAT‐like activity is shown, leading to the decomposition of H_2_O_2_ to produce oxygen and water (Equation 2).^[^ ^15^ ^]^

(1) $$\begin{array}{c} \left(i\right)\;H_{2}O_{2}\to \mathrm{free}\;\mathrm{radicals}\;\left(\mathrm{mainly}\cdot \mathrm{OH}\right)\\ \left(\mathrm{ii}\right)\;\cdot \mathrm{OH}+\mathrm{AH}\to A+H_{2}O\; \end{array}$$

(2) $$2H_{2}O_{2}\to O_{2}+2H_{2}O$$

**FIGURE 2:**
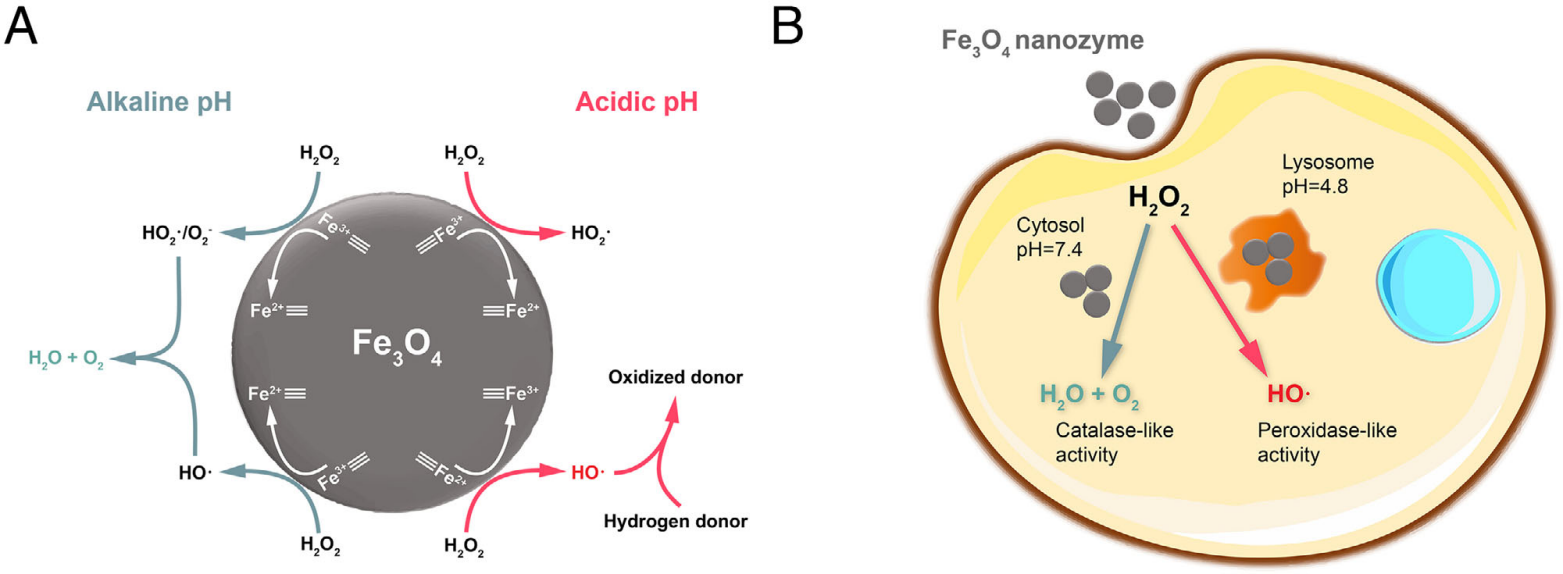
Schematic diagram of the catalytic activity of Fe_3_O_4_ nanozymes in different pH. (A) Reaction mechanism on the surface of Fe_3_O_4_ nanozymes. (B) Two ways Fe_3_O_4_ nanozymes may behave in cells. Adapted with permission.^[^ ^25^ ^]^ Copyright 2016, American Chemical Society

Traditionally, Equation 1 was regarded to be catalyzed only by the Fenton reaction (or Haber‐Weiss reaction) from the release of iron ions in the solution. However, our group first discovered the phenomenon that this process could also be catalyzed by Fe_3_O_4_ NPs itself. Soon it was attributed to the intrinsic POD‐like activity that has not been noticed before.^[^ ^4a^ ^]^ Subsequently, sufficient evidence followed that the catalytic activity was mainly derived from the intact Fe_3_O_4_ NPs, rather than leached iron ions.^[^ ^16^ ^]^ This interesting discovery promoted us and other researchers to further explore the difference between Fe_3_O_4_ nanozymes and the Fenton reaction. The catalytic process of Fe_3_O_4_ nanozymes distinguishes from the Fenton reaction mainly because the former occurs on the surface of crystallized nanostructure, while the latter happens in homogeneous catalytic systems.^[^ ^1^, ^17^ ^]^ In other words, catalysis of Fe_3_O_4_ nanozymes could be identified as a heterogeneous Fenton reaction (Figure 2A).^[^ ^15^ ^]^ Thus, it is easy to understand why the catalytic activity of Fe_3_O_4_ nanozymes depends on multiple factors containing size, morphology, composition, valence, and surface modification, because they affect the surface properties to various degrees, which closely relate to the active sites. It is also intriguing that Fe_3_O_4_ nanozymes are able to mimic some features of natural enzymes, such as following the Michaelis–Menten kinetics and ping‐pong catalytic mechanism as well as showing pH and temperature‐dependent activities.^[^ ^1^ ^]^

Despite the progress made, we admit that the underlying catalytic mechanisms and structural information of nanozymes remain to be fully understood. However, the understanding of the catalytic mechanism of nanozymes (especially Fe_3_O_4_ nanozymes) is occurring step by step. Recently, catalytic kinetics of POD‐mimetic nanozymes were analyzed in detail using density functional theory calculations.^[^ ^18^ ^]^ Moreover, the structure–activity relationship of nanozymes has just been systematically summarized, which will contribute to the *de novo* design of nanozymes overall.^[^ ^7a^ ^]^ Still, researches in this field are an ongoing process and it leaves room for further exploration.

### 2.2 Modulation of cellular oxidative stress: The key to tumor catalytic therapy

ROS are intermediate products that contain oxygen and emerge in the process of intracellular metabolism, mainly containing H_2_O_2_, hydroxyl radicals (·OH), and superoxide anion (O_2_^·–^).^[^ ^19^ ^]^ Homeostasis of reduction/oxidation (redox) level is critical in support of cellular biological functions. At physiological levels, most cells have sufficient capacity to maintain redox level.^[^ ^20^ ^]^ Cell death probably happens when the concentration of ROS exceeds the threshold of redox level. The abnormal metabolism in cancer cells often leads to a high content of H_2_O_2_ in comparison with normal cells,^[^ ^21^ ^]^ which can be applied in various tumor therapies. For instance, chemodynamic therapy (CDT) mainly aims at catalyzing intratumor H_2_O_2_ into ·OH with higher toxicity via the Fenton reaction or Fenton‐like reactions.^[^ ^22^ ^]^ In addition, decomposition of H_2_O_2_ to O_2_ allows for the sensitization of photodynamic therapy (PDT), sonodynamic therapy (SDT), and radiotherapy.^[^ ^23^ ^]^

Nanozymes can also participate in the regulation of cellular redox level, and those with POD‐like activity are able to promote apoptosis in cancer cells via ROS production.^[^ ^24^ ^]^ As for Fe_3_O_4_ nanozymes, which possess pH‐tunable enzyme‐like activities as aforementioned, an exemplary study has revealed its toxic potential. Reported by Chen *et al*., whether IONPs would promote or alleviate the ROS stress of cells significantly depended on their subcellular localization with different pH (lysosomes or cytosol) (Figure 2B).^[^ ^25^ ^]^ For this reason, lysosomes were regarded as the main target of Fe_3_O_4_ nanozyme‐based tumor catalytic therapy.^[^ ^7^, ^26^ ^]^ In addition, though the CAT‐like activity of Fe_3_O_4_ nanozymes is not conducive for ·OH generation, in some cases it could increase the therapeutic efficacy of photosensitizers and sonosensitizers limited by hypoxia in tumor.^[^ ^27^ ^]^ Overall, one of the key issues in current research is to control the intracellular catalytic performance of Fe_3_O_4_ nanozymes to minimize side effects or off‐target consequences.^[^ ^6^ ^]^

### 2.3 Recent advances of Fe_3_O_4_ nanozyme‐based tumor catalytic therapy

Nanomedicine for cancer treatment has attracted much attention in the past decade. Among the frequently used therapies, CDT, which is mediated by ROS generation, is regarded as a promising therapeutic approach owing to its unique advantages in overcoming drug resistance and activating selectively in the tumor microenvironment.^[^ ^28^ ^]^

Tumor catalytic therapy based on ROS generation could be regarded as a novel type of CDT. Increasing researchers are working on the rational design of nanozymes and apply them from theory to practice.^[^ ^8a,b^ ^]^ With POD‐like activity activated by an acidic environment, Fe_3_O_4_ nanozyme is promising as well to contribute to the design of tumor catalytic therapy. A typical work about utilizing Fe_3_O_4_ nanozymes to realize tumor catalytic therapy was reported by Huo *et al*. Through simultaneously encapsulating Fe_3_O_4_ nanozymes and glucose oxidase (GOD) into mesoporous silica, the production of ·OH can be promoted with the help of GOD, which catalyzed glucose into H_2_O_2_.^[^ ^29^ ^]^ In another work designed by An *et al*., the selectivity of Fe_3_O_4_ nanozyme‐based tumor catalytic therapy was augmented via the addition of ascorbic acid, an essential micronutrient to selectively trigger oxidative stress in cancer cells under pharmacological dosage without harming normal cells.^[^ ^7c^ ^]^

**FIGURE 3:**
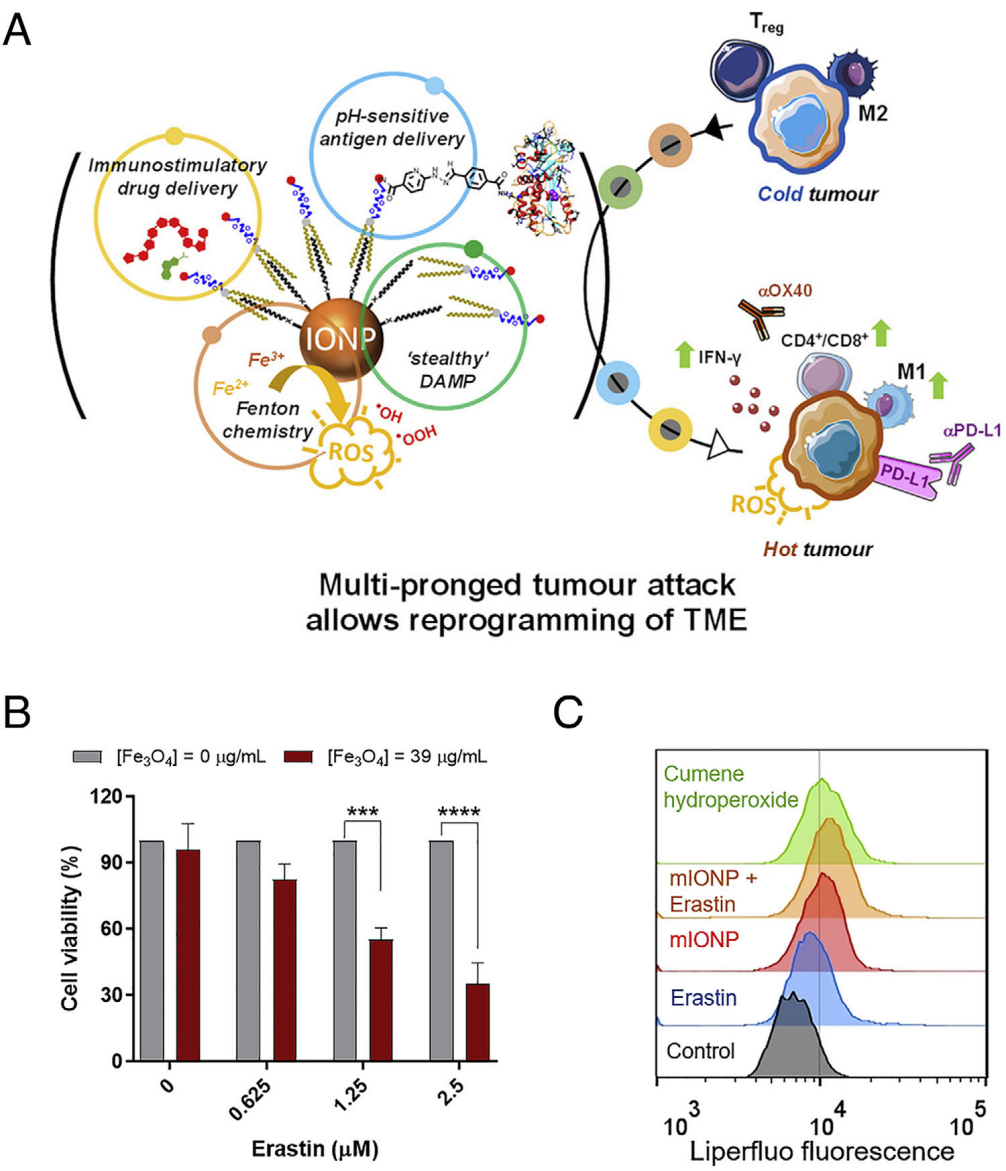
Combination of the POD‐like activity of Fe_3_O_4_ nanozymes with immunotherapy. (A) Graphical abstract of IONVs. (B) Effects of micelles filled with Fe_3_O_4_ nanozymes (mIONPs) on the cell viability of B16‐F10(OVA) cells after 24 h of exposure upon 24 h preconditioning with increasing concentrations of Erastin. Data were normalized to the absence of mIONP treatment for each Erastin concentration. (C) B16‐F10(OVA) cells incubated with mIONPs (1 mM of Fe) for 24 h. Representative flow cytometric profiles demonstrating Liperfluo signals, where cells treated with cumene hydroperoxide are used as positive control. Reproduced with permission.^[^ ^7b^ ^]^ Copyright 2020, Elsevier

Importantly, Fe_3_O_4_ nanozymes also have promising potential to realize enhanced antitumor activity by synergistically coupling with other therapies. According to a recent study, Ruiz‐de‐Angulo *et al*. conjugated ovalbumin (OVA) as antigen and Toll‐like receptor agonists (TLRa) as adjuvant together with micelles filled with Fe_3_O_4_ nanozymes (mIONPs), constructing “nanovaccines” (IONVs) (Figure 3A). They demonstrated that mIONPs with POD‐like activity participated in the induction of ferroptosis. After treating with erastin (a ferroptosis inducing agent) and then incubating with 39 µg/mL of mIONPs, the cell viability of melanoma cells (B16‐F10) significantly decreased (Figure 3B). To further elucidate the occurrence of ferroptosis, the authors evaluated the level of lipid peroxidation harnessing a fluorescent probe (Liperfluo). The flow cytometry analysis showed the presence of hydroperoxide lipids in B16‐F10 cells even without erastin (Figure 3C).^[^ ^7b^ ^]^ In addition, strategies like optimization of the structure and morphology Fe_3_O_4_ nanozymes, doping other metal elements, coupling with photothermal therapy, H_2_O_2_ supplement, and glutathione (GSH) depletion were also developed for the improvement of therapeutic efficacy.^[^ ^7^, ^26^, ^30^ ^]^

### 2.4 Challenges in the development of Fe_3_O_4_ nanozyme‐based tumor catalytic therapy

Although nanozymes synthesized with Fe_3_O_4_ nanoparticles are considered to show great potential for tumor catalytic therapy, no relevant antitumor agents have been approved in clinic (only one commercial product for magnetic fluid hyperthermia).^[^ ^28^, ^31^ ^]^ The existing challenges include the following two points.

First, as an antitumor reagent, the biosafety of Fe_3_O_4_ nanozymes should be carefully examined. Although IONPs have been recognized with favorable biosafety for about nine decades, many relevant commercial products were retracted from the market in the recent years.^[^ ^32^ ^]^ Except for those withdrawals due to poor clinical performance, an important reason came from the varying degrees of adverse effects after intravenous injection in clinical trials and the mechanisms behind are still not completely understood and the release of free iron ions was traditionally regarded as the main cause.^[^ ^3^, ^33^ ^]^ From another perspective, the POD‐like activity of Fe_3_O_4_ nanozymes could be a supplementary explanation. Several studies have reported the POD‐like activity in some FDA‐approved IONPs,^[^ ^9^ ^]^ even though their catalytic activity has been largely assumed to be absent due to passivating coatings.^[^ ^30b^ ^]^ Furthermore, in an animal experiment, the POD‐like activity of systematically injected Fe_3_O_4_ NPs could be detected for at least 3 days by staining the paraffin tissue sections.^[^ ^34^ ^]^ Moreover, we note that the synthesis of most Fe_3_O_4_ NPs for MRI imaging is performed in anaerobic conditions to avoid the loss of magnetic properties.^[^ ^14^ ^]^ In this respect, protecting the oxidation of Fe_3_O_4_ NPs is also associated with the maintenance of Fe_3_O_4_ nanozyme's POD‐like activity since the appropriate ratio of Fe^2+^/Fe^3+^ plays a key role in the catalytic process.^[^ ^18^ ^]^ Taken together, these factors suggest the potential of undesired toxicity *in vivo* once Fe_3_O_4_ nanozymes are localized into environments with relatively low pH and high concentration of H_2_O_2_ (*e.g*. inflammatory microenvironment^[^ ^35^ ^]^), bearing in mind that most of the adverse effects in clinical trials were correlated with immune responses, such as local pain and hypersensitivity.

Second, as for Fe_3_O_4_ nanozyme‐based tumor catalytic therapy, the difficulty in precisely controlling the dual enzyme‐like activities of Fe_3_O_4_ nanozymes is a thorny issue and prevents Fe_3_O_4_ nanozymes from achieving the maximization of therapeutic efficacy. According to the aforementioned mechanism,^[^ ^25^ ^]^ uncertainty of the subcellular localization of Fe_3_O_4_ nanozymes might lead to differences in cellular ROS level, which is highly related to inconsistent outcomes in many studies.

In order to explain these inconsistent outcomes as well as improve the antitumor activity of Fe_3_O_4_ nanozymes, we pay close attention to surface modification owing to its wide range of effects. First, surface modification has powerful influences on the catalytic activity of Fe_3_O_4_ nanozymes.^[^ ^36^ ^]^ Second, the first and most direct interactions happen between surface modification of materials and living systems,^[^ ^37^ ^]^ which will change the physical and chemical properties of Fe_3_O_4_ nanozymes and further affect catalytic activity. Finally, biological responses are largely determined by surface modification at the tissue level and cell level.

## 3 THE IMPORTANCE OF SURFACE MODIFICATION FOR TUMOR CATALYTIC THERAPY

### 3.1 The major classification of surface modification

At nanoscale, unmodified Fe_3_O_4_ nanozymes often have low colloidal stability due to their large surface energy, which makes surface modification almost indispensable for biomedical applications. The importance of surface modification not only lies in providing stability for Fe_3_O_4_ nanozymes in solution. Bearing in mind that nearly all the reactions take place on the surface of Fe_3_O_4_ nanozymes, the catalysis effect, obviously, can be directly affected by those coating materials to some extent. The representative materials for the surface modification of conventional IONPs can be classified into three major categories: polymers, organic small molecules, and inorganic materials (Table 1).

**TABLE 1:** Summary of representative materials for the surface modification of IONPs in accompany with typical applications

Category	Surface modification	Typical application	Reference
Polymer	Poly(ethylene glycol) (PEG)	MRI	[38]
	Dextran	MRI, Magnetic targeted delivery of labeled cells	[39]
	Poly(acrylic acid) (PAA)	MRI	[40]
	Poly(lactic‐*co*‐glycolic acid) (PLGA)	MRI and drug delivery	[41]
	Poly(ethyleneimine) (PEI)^a)^	siRNA delivery	[42]
	Chitosan^a)^	Osteochondral disease diagnosis and reconstruction	[43]
	Distearoyl‐N‐[3‐carboxypropionoyl poly(ethylene glycol) succinyl] phosphatidylethanolamine (DSPE‐PEG)	Photothermal therapy	[44]
Organic small molecules	Citrate	Anti‐platelet therapy	[45]
	Hyperbranched phenylboronic acid	Drug delivery system with pH‐responsive ability	[46]
	Serine	Targeted drug delivery	[47]
	Oleic acid^a)^	Hydrophobic drug delivery	[48]
Inorganic materials	SiO_2_ ^a)^	Loading fluorescent molecules for bioimaging; *in vivo* tracking of labeled cells	[49]
	Carbon^a)^	Drug delivery system with pH‐responsive ability	[50]
	Gold^a)^	Magnetic resonance/photoacoustic dual‐modal imaging	[51]

^a)^ These positively charged polymer and hydrophobic organic/inorganic materials are not suitable for intravenous injection, or they need to be compounded with other materials to improve their hydrophilicity.

Functionalization, additionally, is regarded as another form of surface modification. It means to append new properties to nanoparticles on the basis of colloidally stable nanomaterials. Some representative strategies include grafting fluorescent groups for fluorescence imaging,^[^ ^52^ ^]^ conjugating targeting peptides to target specific sites,^[^ ^53^ ^]^ coating materials sensitive to GSH or pH for stimuli‐responsive release,^[^ ^48^, ^50^, ^54^ ^]^ and covering cell membranes for prolonged circulation.^[^ ^55^ ^]^ Functionalized materials usually have no obvious influence on the catalytic activity of Fe_3_O_4_ nanozymes, but they affect therapeutic efficacy in other ways, such as altering the biodistribution and intracellular fate.

### 3.2 How do surface modifications affect the antitumor activity of Fe_3_O_4_ nanozymes?

#### 3.2.1 Affecting the catalytic activity of Fe_3_O_4_ nanozymes directly

The capability of killing tumor cells of Fe_3_O_4_ nanozymes depends significantly on its POD‐like activity. Generally, major surface modification materials, such as PEG, dextran, SiO_2_, were found to slightly decrease the POD‐like activity of Fe_3_O_4_ nanozymes, probably due to the blocking effect (*e.g*. the active sites on the surface are shielded from interacting with substrates).^[^ ^16^, ^36^ ^]^ A good example for explaining the blocking effect is that the POD‐like activity of Fe_3_O_4_ nanozymes was significantly enhanced by etching and removing the SiO_2_ shell to form a yolk‐shell structure during the synthesis process.^[^ ^56^ ^]^ Moreover, encapsulating with mesoporous silicon could also avoid the blocking effect probably due to the increase of the porosity of SiO_2_ shell.^[^ ^26b^ ^]^

It is also a general rule that surface modification materials with different surface charges may enhance the POD‐like activity towards different substrates. When taking anionic ABTS as the substrate, Fe_3_O_4_ nanozymes modified with positively charged materials, such as PLL (poly‐l‐lysine) and PEI, showed higher catalytic activity, while negatively charged materials, such as citrate and dextran, were in favor of the oxidation of the cationic substrate TMB.^[^ ^36^ ^]^

In the field of chemical catalysis, researchers have paid efforts to improve the POD‐like activity of Fe_3_O_4_ nanozymes effectively. A typical strategy is to mimic the natural enzymatic microenvironment of peroxidase by modifying single amino acids, such as histidine, whose imidazole group assists the location of H_2_O_2_ into the active site via H‐bond interactions.^[^ ^57^ ^]^ In another work, Zhang *et al*. constructed substrate‐binding pockets by molecular imprinting.^[^ ^58^ ^]^ The key of these two strategies lies in improving the affinity and selectivity of substrates. Besides, the range of reaction pH could be widened from 3–4 to 3–9 thanks to sulfidation.^[^ ^59^ ^]^ However, most of these advances have not been tested in a biological environment. Whether these modification strategies are able to enhance the therapeutic efficacy remains to be explored in the future.

#### 3.2.2 Affecting the catalytic activity of Fe_3_O_4_ nanozymes indirectly

In addition to directly affecting the catalytic process, surface modification also indirectly affects the catalytic activity of Fe_3_O_4_ nanozymes, which is also worth noting. Due to the complicated environment in vivo, interactions (mainly including protein corona adsorption, aggregation, and degradation) often happen between surface modification materials and biomolecules. For example, protein corona adsorption and aggregation are related to activation/inhibition of the active sites of Fe_3_O_4_ nanozymes, and degradation is in connection with the integrity of the nanostructure of Fe_3_O_4_ nanozymes. Research progresses on these three interactions are introduced in this section.

Protein corona adsorption means that the catalytic activity can be regulated by molecules from biological environment. Protein corona (PC) is the proteins adsorbed on the surface of nanomaterials in a physiological solution through electrostatic interactions and hydrophobic interactions. PC can be classified into hard corona and soft corona according to the property of adsorbed proteins. Hard corona refers to those binding with nanoparticles tightly and irreversibly, while soft corona binds with nanoparticles weakly and dynamically.^[^ ^60^ ^]^ Since the formation of PC could almost be recognized as the second surface modification of nanoparticles, PC‐adsorbed surfaces are quite different from the original ones, which have attracted attention in the field of nanomedicine.^[^ ^3^, ^60^, ^61^ ^]^

Generally, the effect of PC on the catalytic activity of nanozyme depends not only on the composition and quantity of PC, but also on the accessibility of different substrates to the surface of nanozymes. For instance, Zhang *et al*. lately reported that how the different formations of hard/soft corona influenced the activity of ruthenium‐based nanozymes carried by Au NPs (abbreviated as NZ). It was reported that a “supramolecular gate” formed around NZ1 (binding hard corona) to block the access of substrates, hence decreasing the catalytic activity. In contrast, NZ2 (binding soft corona) reserved part of the catalytic activity. Subsequently, reactivation was detected both in Corona‐NZ1 and Corona‐NZ2. The reason lied in the degradation of PC by proteases in lysosomes.^[^ ^62^ ^]^ As for Fe_3_O_4_ nanozymes, the adsorption of proteins may block the active sites on their surface.^[^ ^36^ ^]^ However, Wang *et al*. reported that hard corona surprisingly increased the POD‐like activity of Fe_3_O_4_ nanozymes via its different functional groups, conformations, and protein surface charge, which would be beneficial for the rational design of Fe_3_O_4_ nanozyme‐based nanomedicine.^[^ ^63^ ^]^

There is a close relationship between surface modification and the formation of PC. First, a hard corona is prone to form on a hydrophobic surface owing to the denaturation and irreversible adsorption of proteins, whereas a soft corona is formed without hydrophobic interactions.^[^ ^62^ ^]^ Second, nanoparticles with a higher surface charge tend to adsorb a larger amount of PC than those with a neutral surface charge in general and positively/negatively charged nanoparticles differ greatly in the composition of PC.^[^ ^64^ ^]^ Third, the density and molecular weight of modification materials also have a comprehensive impact on PC's composition and quantity.^[^ ^65^ ^]^

But as for the antitumor activity, more details should be noted. Apart from affecting the catalytic activity of Fe_3_O_4_ nanozymes, PC is able to influence other physiological processes such as increasing or inhibiting cellular uptake, hindering active targeting or contributing to passive targeting, and stimulating or mitigating immune responses.^[^ ^61^ ^]^ In addition, the difference in experimental conditions should be taken into consideration. For example, Wang *et al*. reported nontoxicity of Fe_3_O_4_ NPs to cancer stem cells (which were derived from human glioblastoma cell line U251). Furthermore, they found that Fe_3_O_4_ NP‐mediated ROS generation accelerated the cell cycle progression.^[^ ^66^ ^]^ On one hand, it might be due to the different metabolism of stem cell lines.^[^ ^9c^ ^]^ On the other hand, it is worth noting that the cancer stem cells were re‐suspended in a serum‐free medium.^[^ ^66^ ^]^ Similarly, Fe_3_O_4_ NPs were reported to show CAT‐like activity in PC12 cells and caused reduced toxicity.^[^ ^67^ ^]^ While PC12 cells were cultured with 10% heat‐inactivated serum, which was found to alter cellular uptake because complement proteins would be denatured after heat‐inactivation.^[^ ^68^ ^]^ In summary, there are still unknowns in our understanding of the effect of PC on Fe_3_O_4_ nanozyme's antitumor activity and it deserves further investigation.

The aggregation of Fe_3_O_4_ nanozymes has been proved to inhibit the POD‐like activity.^[^ ^69^ ^]^ The surface chemistry of some surface modification materials is changed after interacting with a biological environment, such as the protonation of ammonium group and the adsorption of protein corona,^[^ ^70^ ^]^ hence improving or inhibiting the aggregation of nanoparticles. It can be speculated that the POD‐like activity of Fe_3_O_4_ nanozymes is able to be inactivated/reactivated through aggregation/dispersion.

The degradation of Fe_3_O_4_ nanozymes is highly related to the interaction between surface modification materials and the endosomal/lysosomal environment. As a representative modification material, dextran is degradable through hydrolysis by α‐glucosidase in lysosomes.^[^ ^71^ ^]^ Additionally, dicarboxylic acid (e.g., citrate and isocitrate) in lysosomes acts as chelators to help with the release of iron ions from the iron oxide cores.^[^ ^72^ ^]^ Therefore, these factors accelerate the degradation rate of IONPs in cells. Furthermore, the rate of degradation could be modulated by the thickness of modified carboxydextran shells. In a comparative experiment of two IONPs sharing the same size of iron oxide cores but with different thickness of carboxydextran shells, the thinner carboxydextran shell on the surface of IONPs resulted in a more rapid degradation, allowing quicker ROS generation and a stronger cytotoxicity.^[^ ^73^ ^]^ Mazuel *et al*. compared the degradation extent of citrate molecule modified γ‐Fe_2_O_3_ NPs and polymer‐modified γ‐Fe_2_O_3_ NPs in the endosome/lysosome of hMSCs. Essentially due to the protection of polymeric coating, only 20% of the polymer‐modified γ‐Fe_2_O_3_ NPs were degraded after 27 days. However, almost all citrate‐modified γ‐Fe_2_O_3_ NPs were degraded (>90%), on account of the weak resistance of citrate as a small molecule to the lysosome environment.^[^ ^74^ ^]^ This result also explained why the POD‐like activity of ferumoxytol (a type of IONPs modified with polymer carboxymethyl‐dextran) was preserved better than the citrate‐coated IONPs.^[^ ^9a^ ^]^

At present, whether the degradation of Fe_3_O_4_ nanozymes is in favor of the overall ROS generation has not reached a definite conclusion. According to some studies, a rapid release of iron ions from Fe_3_O_4_ NPs was also a strategy to realize tumor killing via the Fenton reaction or other ion‐mediated catalytic mechanisms.^[^ ^75^ ^]^ However, decomposition of the iron oxide cores will end up with the loss of Fe_3_O_4_ nanozymes’ catalytic activity, which is not conducive for a prolonged treatment.^[^ ^27a^ ^]^ In conclusion, how to keep the balance between the sustainability of the treatment and the biodegradability of Fe_3_O_4_ nanozymes is a challenging task for the design of Fe_3_O_4_ nanozyme‐based tumor catalytic therapy.

Taken together, the relationship between antitumor activity and PC adsorption, aggregation, and degradation in the context of Fe_3_O_4_ nanozymes has yet to be investigated comprehensively *in vivo*. However, according to the literature, a change in catalytic activity usually shows an influence on antitumor activities.^[^ ^7^, ^26^ ^]^ If we hope to design Fe_3_O_4_ nanozymes with optimized catalytic activity for tumor therapy in clinic, more attention on their interactions with living systems is required.

#### 3.2.3 Affecting the biodistribution of Fe_3_O_4_ nanozymes

Upon intravenous injection of Fe_3_O_4_ nanozymes, their biodistribution will be affected by surface modification mainly from two aspects—tumor targeting and in vivo clearance.

It is well‐known that cellular uptake of IONPs with positive surface charge will be promoted because of Coulombic interactions with negatively charged cellular membranes,^[^ ^76^ ^]^ which will enhance the cytotoxicity to cancer cells. Furthermore, functionalization with targeting components, such as cancer‐targeting antibody, is another important strategy for the enhancement of cellular uptake.^[^ ^77^ ^]^ Cancer cell membrane camouflage, in addition, is advisable to improve tumor targeting ability of Fe_3_O_4_ nanozymes due to the homotypic targeting ability produced by the retained cell membrane surface proteins.^[^ ^78^ ^]^

In addition, in vivo clearance should also be taken into consideration. For instance, though positively charged nanomaterials are effective for cellular uptake *in vitro*, they tend to be readily captured by reticuloendothelial system (RES) during blood circulation. Therefore, intravenously injected Fe_3_O_4_ nanozymes should have a neutral to mild negative surface charge to avoid rapid clearance.^[^ ^79^ ^]^ Even so, Fe_3_O_4_ nanozymes are still prone to passively accumulate in the liver due to the abundant phagocytic cells there as well as the holes with sizes of 50–200 nm in the liver sinusoids.^[^ ^80^ ^]^ In fact, such accumulation allows for some FDA approved MRI contrast agents for liver imaging.^[^ ^81^ ^]^ However, it also becomes the reason of hepatotoxicity and is adverse for tumor therapy.^[^ ^3^ ^]^ In this respect, PEG is commonly leveraged by researchers to endow nanomaterials with a “stealth” property in circulation. However, PEGylation was proved to induce the production of anti‐PEG antibodies in humans, which may severely restrict its clinical translation.^[^ ^82^ ^]^ Besides, cell membranes and liposomes are also regarded as suitable materials with high biosafety and long‐circulating ability.^[^ ^55^ ^]^

#### 3.2.4 Affecting the intracellular fate of Fe_3_O_4_ nanozymes

In order to realize tumor catalytic therapy, Fe_3_O_4_ nanozymes must be internalized by cells to exert their catalytic activity. The intracellular fate after internalization is more complicated than the aforementioned processes because it might be related to multiple factors sometimes. Relevant studies are summarized in Table 2 and analyzed below.

**TABLE 2:** Summary of the influence on the intracellular fate of Fe_3_O_4_ nanozymes with different surface modification (in company with other parameters)

Classification	Name	Surface modification	Hydrodynamic size (nm) and zeta Potential (mV)	Cell line	Sample concentration and incubation time	Biological effects	Ref.
Lysosome/cytosol localization	Fe_3_O_4_ NPs	None	45 nm in water/200 nm in complete medium and n.d.	L929 cells and PC12 cells	0‐200 µg/mL and 0–24 h	Reduced ROS levels to protect cells, alleviated neurodegeneration, and increased longevity of AD Drosophila	^[^ ^11^ ^]^
	Fe_3_O_4_ NPs	None	10‐15 nm and n.d.	HeLa cells, 293T cells, L6 cells, HepG2 cells and 3T3‐L1 cells	0‐50 µg/mL and 0–48 h	Fe_3_O_4_ NPs in lysosomes induced local elevation of ROS level and triggered AMPK activation, but Fe_3_O_4_ NPs in cytosol induced ROS scavenging at the whole level	^[^ ^8c^ ^]^
	Fe_3_O_4_@Dex‐TPP/PpIX/ssmPEG	Dextran‐TPP,PpIX‐COOH, and mPEG‐ss‐COOH	76.91±5.33 nm (in PBS) and ‐5.73±0.65 mV	4T1 cells	0‐200 µg/mL and 0–24 h	A small number of cells dead via Fenton reaction without laser irradiation, and a large number of cells dead laser irradiation when the PpIX concentration was over 2.5 µg/mL	^[^ ^27b^ ^]^
Autophagy	PEI‐Fe_3_O_4_ NPs	PEI	26.3±4.42 nm and +25.1±1.23 mV	Hela cells, MCF‐7 cells, HepG2 cells	40 µg/mL and 24 h	Induced autophagy, activated nf‐κb and TGF‐β signaling pathways, escalated ROS production but did not induce acute cytotoxicity	^[^ ^86^ ^]^
	Fe_3_O_4_ NPs and α‐Fe_2_O_3_ NPs	Carboxylic acid	220 and 273 nm in complete medium and n.d.	PC12 cells	0‐200 µg/mL and 0–48 h	Damage to mitochondria, reduced cell viability, and severe autophagy were caused by α‐Fe_2_O_3_ NPs (but not Fe_3_O_4_ NPs)	^[^ ^93^ ^]^
	SPION modified with PEG (SMG) or PEI (SEI)	PEG and PEI	SMG‐10/30: 16.5±4.7 nm/35.8±10.3 nm and ‐0.52 mV/‐0.52 mV; SEI‐10: 17.2±5.0 nm and +29.28 mV	SKOV‐3 cells and RAW 264.7 cells	0‐100 µg/mL and 0–48 h	SEI‐10 induced higher intracellular ROS generation and cytotoxicity; SMG‐10 and SMG‐30, but not SEI‐10, significantly increased autophagy level	^[^ ^79^ ^]^
Cell lethality	Fe_3_O_4_ NPs	OA‐DSPE‐PEG‐NH_2_	30 nm and ‐30 mV	Cancer cells (mainly HepG2 cells)	0‐300 µg/mL and 6–72 h	Induced cell death with features like massive accumulation of cellular vesicles, swelling mitochondria and lysosome, distinct chromatin condensation, and margination	^[^ ^7a^ ^]^
Exocytosis	Citrate‐Fe_3_O_4_ NPs, Fe_3_O_4_@SiO_2_ NPs	Citrate and SiO_2_	Citrate‐Fe_3_O_4_ NPs: 6.34 nm and ‐28.5 mV Fe3O4@SiO2 NPs: 43.24 nm and ‐40.0 mV	hMSCs	100 µg [Fe]/mL and 0–14 days	Citrate‐Fe_3_O_4_ NPs were degraded in lysosomes; Fe_3_O_4_@SiO_2_ NPs were stable and were almost excreted in the first 3 days	^[^ ^91^ ^]^

*Notes*:.

(1) Unless particularly stated, the hydrodynamic size and zeta potential were measured in water.

(2) Sample concentration & incubation time mainly accord to cytotoxicity assays.

**FIGURE 4:**
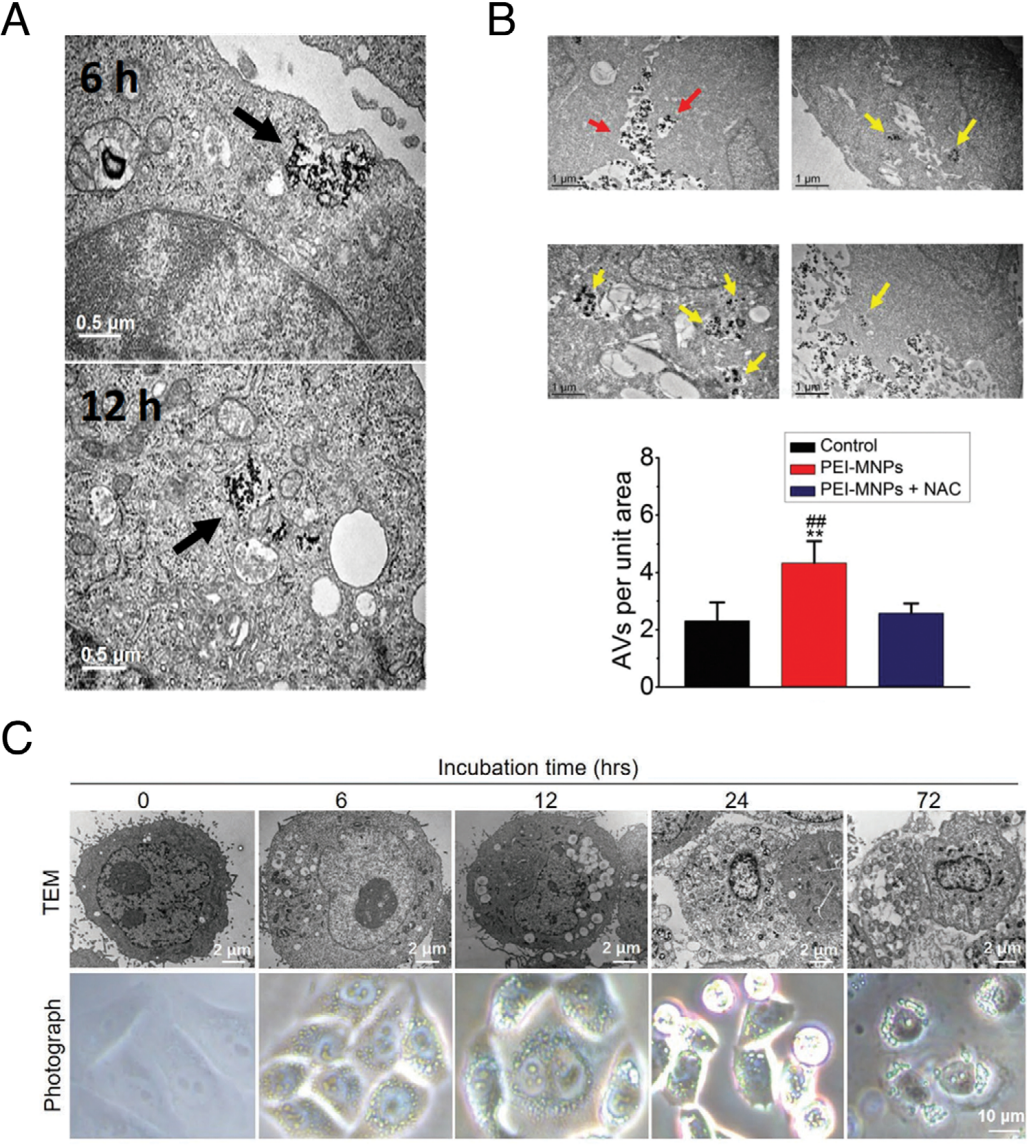
Different intracellular fates affected by surface modification. (A) Internalization of Fe_3_O_4_ NPs in L929 cells at indicated time of exposure (Fe_3_O_4_ NPs were localized in cytoplasm after 12 h). Reproduced with permission.^[^ ^11^ ^]^ Copyright 2016, Wiley Online Library. (B) PEI‐MNPs treatment caused more accumulation of autophagic vacuoles in cancer cells. Reproduced with permission.^[^ ^86^ ^]^ Copyright 2020, Royal Society of Chemistry. (C) Visualization of the morphology of HepG2 cells treated over time with Fe_3_O_4_ NPs (Fe_3_O_4_ NPs were localized in lysosomes). Reproduced with permission.^[^ ^7a^ ^]^ Copyright 2020, Wiley Online Library

Localization in endosome/lysosome or cytosol plays a critical role.^[^ ^25^, ^83^ ^]^ According to Fan and Song's group, Fe_3_O_4_ nanozymes are localized in the cytosol rather than in endosomes/lysosomes (Figure 4A).^[^ ^11^ ^]^ Bearing in mind that Fe_3_O_4_ nanozymes exhibits CAT‐like activity in neutral pH, which is consistent with the pH in cytosol, it seems logical to explain the decomposition of intracellular H_2_O_2_ into H_2_O and O_2_, thus leading to increased longevity of Drosophila. In a recent study, their group further observed that still a fraction of Fe_3_O_4_ nanozymes existed in lysosomes, exhibiting POD‐like activity to produce ROS locally, but the majority of the Fe_3_O_4_ nanozymes in the cytosol performed the ROS scavenging function.^[^ ^8c^ ^]^ Thus, there are two possible explanations: (**i**) this localization behavior was due to the unmodified surface of Fe_3_O_4_ nanozymes, which might penetrate cell membranes—like a previous work on platinum‐decorated ceria nanoparticles;^[^ ^84^ ^]^ (**ii**) most of the Fe_3_O_4_ nanozymes were able to escape from endosomes/lysosomes after endocytosis. Further work is still needed to fully understand the underlying mechanisms and processes.

Inspired by the lysosomal‐escape phenomenon, Hou *et al*. designed a near‐infrared (NIR) laser assistant strategy to enhance the efficiency of lysosomal escape. In this work, Fe_3_O_4_ NPs were modified with a mitochondrial‐targeting group triphenylphosphine (TPP) and a photosensitizer protoporphyrin IX (PpIX), and then they were coated with GSH‐responsive PEG (ssmPEG) forming Fe_3_O_4_@Dex‐TPP/PpIX/ssmPEG. This hybrid nanozyme was endocytosed and localized at lysosomes, and the ssmPEG on the outer layer protected TPP from exposure. During this period, the lysosomal environment allowed the Fe_3_O_4_ nanozymes to generate ·OH. With irradiation by an NIR laser, the lysosomal membranes were damaged by ^1^O_2_ from PpIX and caused lysosomal escape. The high content of GSH in the cytosol of 4T1 cancer cells led to the cleavage of ssmPEG and the exposed TPP consequently guided the Fe_3_O_4_ nanozymes to mitochondria to exert CAT‐like activity, producing O_2_ for PpIX‐mediated ^1^O_2_ generation.^[^ ^27b^ ^]^ The precise intracellular localization controlled by surface modification and external stimuli took full advantage of the dual enzyme‐like activities of Fe_3_O_4_ nanozymes.

Apart from endosomes/lysosomes, autophagosomes are another major subcellular structure that usually form in response to many kinds of nanoparticles.^[^ ^70b^ ^]^ Wang *et al*. observed α‐Fe_2_O_3_ NPs and Fe_3_O_4_ NPs modified with carboxylic acids caused varying degrees of autophagy in PC12 cells. After treatment with an autophagy inhibitor 3‐methyladenine, they found the viability in NP‐treated PC12 cells was partially rescued.^[^ ^67^ ^]^ It could be speculated that the POD‐like activities of α‐Fe_2_O_3_ NPs and Fe_3_O_4_ NPs were enhanced due to the fusion of autophagosomes and lysosomes, which lowered the environmental pH. In addition, α‐Fe_2_O_3_ NPs performed higher POD‐like activity and therefore induced autophagy more significantly than Fe_3_O_4_ NPs according to their experiments, which was likely due to the higher CAT‐like activity of Fe_3_O_4_ NPs compared to α‐Fe_2_O_3_ NPs.^[^ ^67^ ^]^

In comparison with negatively charged surface modification materials, positively charged materials are prone to induce a higher degree of autophagy in various cell lines.^[^ ^85^ ^]^ PEI is one of the most investigated cationic polymers. Gao *et al*. confirmed that PEI could trigger lysosomal‐escape and subsequently induce autophagy in cells.^[^ ^85a^ ^]^ A similar phenomenon was also observed by Man *et al*. Using PEI‐modified Fe_3_O_4_ NPs (PEI‐MNPs) up to 40 µg/mL, which was a relatively low concentration, the cellular viability was negligibly affected, but the ROS level and autophagy level were significantly escalated (Figure 4B), which would probably contribute to the design of antitumor strategies.^[^ ^86^ ^]^ Nonetheless, an inconsistent result was found by Feng *et al*., wherein PEG modified Fe_3_O_4_ NPs induced significant autophagy but PEI modified Fe_3_O_4_ NPs did not.^[^ ^79^ ^]^ Therefore, an in‐depth understanding of autophagy is also valuable for analyzing the underlying toxicological mechanisms.

In particular, Wang *et al*. recently reported that Fe_3_O_4_ nanozymes induced significant cell death via ROS generation under the acidic lysosomal environment.^[^ ^7a^ ^]^ Importantly, this lethal effect was proved to be independent of free iron after the addition of ferroptosis inhibitor ferrostatin‐1. Herein, the phenomenon of lysosomal swelling is worth noting because it may directly relate to the POD‐like activity of Fe_3_O_4_ nanozymes (Figure 4C). According to the reagents used in the experiments in this paper, the iron oxide cores were modified with oleic acid (OA) and the OA layer was further conjugated with DSPE‐PEG with an amino‐terminal (DSPE‐PEG‐NH_2_).^[^ ^7a^ ^]^ The OA layer was possible to protect the Fe_3_O_4_ nanozymes from inactivation because some anions (like PO_4_^3–^ and CO_3_^2–^) in biological solution are proved to adsorb on the active sites (especially Fe(**II**)) and inhibited the POD‐like activity, which could be reduced by the hydrophobic inner layer.^[^ ^87^ ^]^ In contrast, Fe_3_O_4_ NPs modified with dimercaptosuccinic acid (DMSA) and citrate were generally not toxic to cells,^[^ ^10a^ ^,^ ^88^ ^]^ probably because these hydrophilic small molecules were difficult to protect the surface of Fe_3_O_4_ NPs from adsorption of PO_4_^3–^ and CO_3_^2–^. This effect was also confirmed by Ruiz‐de‐Angulo *et al*. They performed control experiments between IONPs (modified with DSPE‐cPEG(2000)‐phospholipid) and Ferumoxytol (Fe_3_O_4_ NPs modified with carboxydextran). The former exhibited a higher POD‐like activity and more potent inhibition of tumor growth than the latter.^[^ ^7b^ ^]^ In addition, the PC formed on the surface of NH_2_‐terminal PEG might promote the cellular uptake of Fe_3_O_4_ nanozymes.^[^ ^89^ ^]^ Furthermore, the protonation of the amino group perhaps was related to lysosomal swelling.^[^ ^70a^ ^]^ However, these analyses are still in need of further experimental verification.

Exocytosis, in general, is regarded to be against therapeutic efficacy. For instance, according to Gu *et al*., exocytosis might be initiated by RAW264.7 cells to reduce the concentration of intracellular γ‐Fe_2_O_3_ NPs so that the cytotoxicity was alleviated.^[^ ^90^ ^]^ Such a phenomenon was particularly evident when hMSCs internalized non‐degradable SiO_2_‐coated Fe_3_O_4_ NPs.^[^ ^91^ ^]^ Conversely, an exocytosis‐inhibition strategy gained positive outcomes in tumor therapy by using polydopamine modified mesoporous silica as the modification material.^[^ ^92^ ^]^ This result suggested that selectively overcoming exocytosis might be helpful to Fe_3_O_4_ nanozyme‐based tumor catalytic therapy.

## 4 CONCLUSIONS AND PERSPECTIVE

**FIGURE 5:**
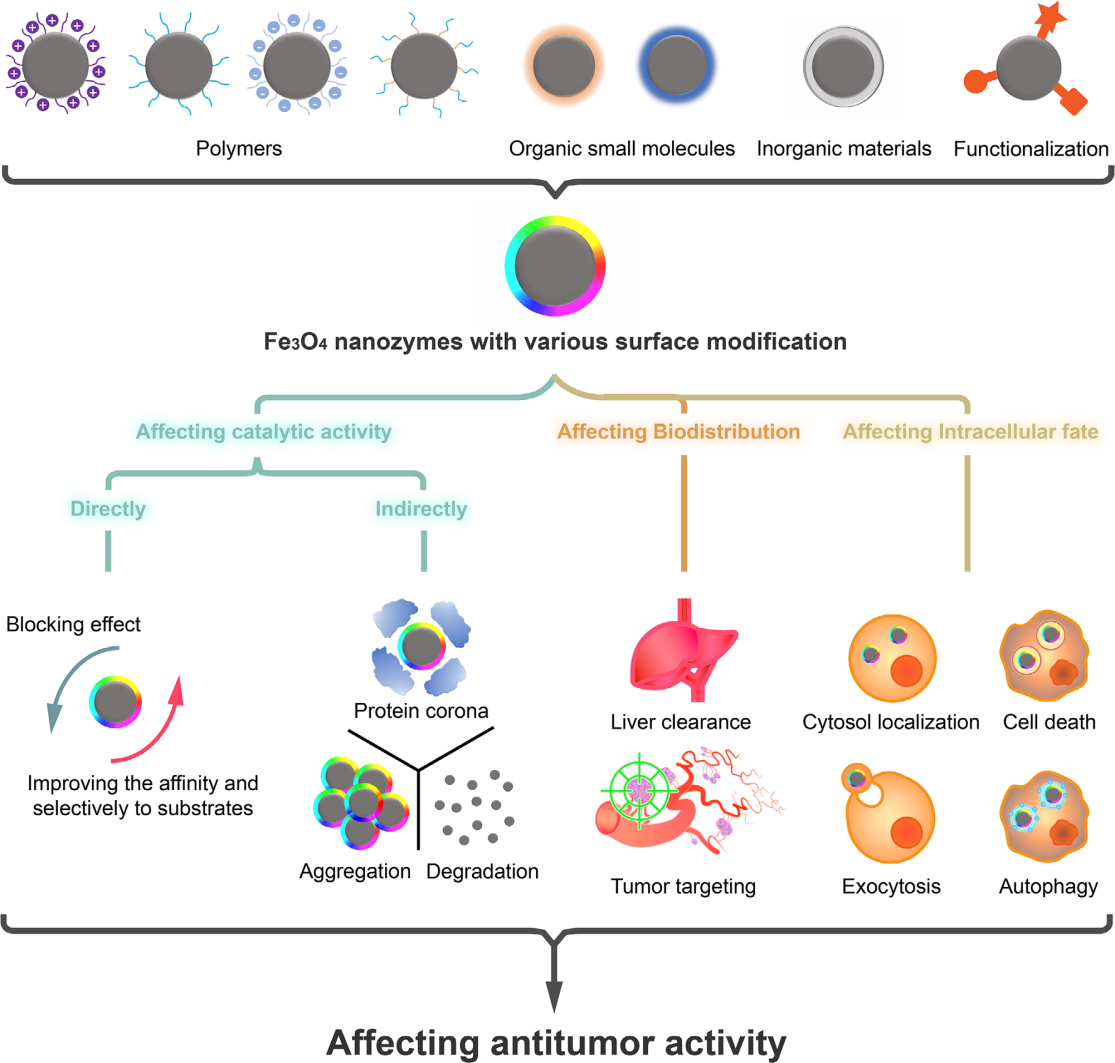
Schematical illustration of the ways of surface modification to affect the antitumor activity of Fe_3_O_4_ nanozymes

Altogether, this review highlights the relationship between the antitumor activity and surface modification of Fe_3_O_4_ nanozymes (schematically illustrated in Figure 5). On one hand, multiple factors such as surface charge, hydrophilicity/hydrophobicity, and thickness of surface modification materials need to be considered. On the other hand, advanced technologies are necessary to track nanoparticles in living cells with a high spatiotemporal resolution,^[^ ^70a^ ^,^ ^83^ ^]^ and it is also important to precisely monitor real‐time changes of the redox levels in specific organelles.^[^ ^94^ ^]^ Herein, we also provide some strategies of surface modification with the hope to improve the antitumor performance of Fe_3_O_4_ nanozymes.

### 4.1 Improving the biosafety of FDA‐approved IONPs and the selectivity of antitumor activity

Similar to CDT, Fe_3_O_4_ nanozyme‐based tumor catalytic therapy is recognized to show a certain degree of selectivity to cancer cells mainly due to the relatively higher concentration of H_2_O_2_. Nevertheless, the selectivity of Fe_3_O_4_ nanozymes has plenty of room for improvement.^[^ ^7c^ ^]^ On this basis, strategies such as augmenting Fe_3_O_4_ nanozymes’ catalytic activity by in situ near‐infrared laser irradiation or encapsulating Fe_3_O_4_ nanozymes into carriers with tumor microenvironment triggered release capability have been designed.^[^ ^95^ ^]^ Moreover, by means of specific surface modification, it could be feasible to inhibit the POD‐like activity of Fe_3_O_4_ nanozymes during the delivery process, and then activate it at the tumor site in a stimuli‐responsive manner. This strategy is also promising to reduce side effects and systematic toxicity, hence improving the biosafety of FDA‐approved IONPs.

### 4.2 Targeting tumor‐associated immune cells

In some cases, biodistribution in non‐tumor cells can also be utilized to exert antitumor activity. Korangath *et al*. observed that the retention of antibody‐labeled Fe_3_O_4_ NPs in orthotopic mammary tumors not only depended on the interaction between tumor cells and antibodies, but also was determined by tumor‐associated immune cells (dendritic cells, neutrophils, monocytes, and macrophages) to a great extent.^[^ ^96^ ^]^ According to another work, Zanganeh *et al*. used ferumoxytol (Fe_3_O_4_ NPs modified with carboxymethyldextran) to kill cancer cells in an indirect manner: inducing polarization of tumor‐associated macrophages (TAMs) for immunotherapy. It was confirmed that ferumoxytol caused macrophage polarization into pro‐inflammatory M1 phenotypes via the Fenton reaction.^[^ ^30b^ ^]^ Still, we need to be careful of the dose‐dependent cytotoxicity caused in normal immune cells.^[^ ^71^, ^97^ ^]^ The specifical targeting ability to TAMs was improved by Liu *et al*. in a current study, in which Fe_3_O_4_ NPs and other components were co‐encapsulated into lipopolysaccharide‐treated macrophage membranes.^[^ ^98^ ^]^ These results suggested a new idea that targeting tumor‐associated immune cells could be another feasible surface modification strategy.

### 4.3 Improving the precision of subcellular localization

In general, the acidic environment in lysosomes or autolysosomes is appropriate for Fe_3_O_4_ nanozymes to exert POD‐like activity before degradation. In other words, maybe it is suitable for surface modification strategies to aim at lysosomal retention. As for PDT and photothermal therapy (PTT), mitochondria are ideal targets for destruction due to their important role in the redox balance of cells.^[^ ^27^, ^99^ ^]^ Exocytosis, according to recent advances in drug delivery, might not always go against therapeutic efficacy. As a matter of fact, it could be leveraged to realize transcytosis via charge‐reversal surface modification, hence improving deep tu,mor penetration.^[^ ^100^ ^]^

To sum up, there is still ample room for the development of Fe_3_O_4_ nanozyme‐based tumor catalytic therapy. Although various nanomaterials have been extensively studied through *in vitro* cell models and animal models, many open gaps still exist in our current understanding about what happens to the surface modification materials in real time. As for Fe_3_O_4_ nanozymes, it is still challenging to stimulate their interactions with the biological environment perfectly *in vitro*. In some cases, variations of the surface modification materials *in vivo* would be far from our exception during long‐term metabolism. Therefore, continuous investigation is urgently needed in this area. Hopefully, figuring out the influence of surface modification in depth will help the precise control of Fe_3_O_4_ nanozyme's catalytic performance in cells, which will also pave its way from bench to bedside.

## AUTHOR CONTRIBUTIONS

Guoheng Tang and Jiuyang He contributed equally to this work.

## CONFLICT OF INTEREST

The authors declare no conflict of interest.
